# Proteolytic Pathways Induced by Herbicides That Inhibit Amino Acid Biosynthesis

**DOI:** 10.1371/journal.pone.0073847

**Published:** 2013-09-06

**Authors:** Amaia Zulet, Miriam Gil-Monreal, Joji Grace Villamor, Ana Zabalza, Renier A. L. van der Hoorn, Mercedes Royuela

**Affiliations:** 1 Departamento de Ciencias del Medio Natural, Universidad Pública de Navarra, Pamplona, Spain; 2 Plant Chemetics Laboratory, Max-Planck Institute for Plant Breeding Research, Cologne, Germany; Iwate University, Japan

## Abstract

**Background:**

The herbicides glyphosate (Gly) and imazamox (Imx) inhibit the biosynthesis of aromatic and branched-chain amino acids, respectively. Although these herbicides inhibit different pathways, they have been reported to show several common physiological effects in their modes of action, such as increasing free amino acid contents and decreasing soluble protein contents. To investigate proteolytic activities upon treatment with Gly and Imx, pea plants grown in hydroponic culture were treated with Imx or Gly, and the proteolytic profile of the roots was evaluated through fluorogenic kinetic assays and activity-based protein profiling.

**Results:**

Several common changes in proteolytic activity were detected following Gly and Imx treatment. Both herbicides induced the ubiquitin-26 S proteasome system and papain-like cysteine proteases. In contrast, the activities of vacuolar processing enzymes, cysteine proteases and metacaspase 9 were reduced following treatment with both herbicides. Moreover, the activities of several putative serine protease were similarly increased or decreased following treatment with both herbicides. In contrast, an increase in YVADase activity was observed under Imx treatment versus a decrease under Gly treatment.

**Conclusion:**

These results suggest that several proteolytic pathways are responsible for protein degradation upon herbicide treatment, although the specific role of each proteolytic activity remains to be determined.

## Introduction

Herbicides that inhibit amino acid biosynthesis are useful tools in weed management and have been particularly successful because of their low toxicity in mammals, as these herbicides inhibit pathways that are lacking in mammals. There are several types of herbicides whose targets or primary sites of action are associated with the specific inhibition of enzymatic activity in biosynthetic pathways for amino acids. One such group of herbicides comprises compounds that inhibit the biosynthesis of branched-chain amino acids (valine, leucine and isoleucine) through the inhibition of acetolactate synthase (ALS, EC 4.1.3.18), also referred to as acetohydroxyacid synthase. ALS inhibitors include the active ingredients of several classes of chemicals and have become one of the most widely used types of herbicides because of their wide-spectrum weed control activity, high crop selectivity, low required application rates and low toxicity in mammals [1]. Glyphosate (Gly) is another type of herbicide that inhibits amino acid biosynthesis, through inhibition of 5-enolpyruvylshikimate-3-phosphate synthase (EPSPS, EC 2.5.1.19) [2], which is involved in the biosynthesis of aromatic amino acids (tyrosine, phenylalanine and tryptophan). Gly is a wide-spectrum, non-selective post-emergence herbicide that is the most popular herbicide used worldwide, particularly since the introduction of transgenic Gly-resistant crops [3].

Although the targets (mechanisms of action) of these two types of herbicides are well known, it is not fully understood what causes plant death following the inhibition of ALS or EPSPS. Several physiological effects in the mode of action of ALS and EPSPS inhibitors have been described. Interestingly, most of these effects are common, although the target sites involved are different. A general physiological effect reported following both EPSPS and ALS inhibition is growth arrest, followed by the slow death of treated plants [4], [5]. Carbon metabolism is impaired following the application of both types of herbicides, while aerobic fermentation in roots is induced [6], [7], and the carbohydrate content of roots and leaves is increased upon treatment [7], [8]. The increased sucrose and starch content detected in the roots following herbicide treatment triggers a decrease in sink strength, which inhibits phloem transport and causes carbohydrate accumulation in the leaves [8]. Another common physiological effect observed after the use of these classes of herbicides is accumulation of secondary metabolites, such as quinate, a compound synthesized in a lateral branch of the shikimate pathway [9].

Additional specific common biochemical effects of ALS and EPSPS inhibitors are an increase in the free amino acid pool and a decrease in the soluble protein content. Amino acid accumulation has been observed in pea plants treated with lethal doses of Gly [7], [9]. Similarly, an increase in free amino acid contents following ALS inhibition have been widely reported [9]–[14]. Furthermore, a decrease in the amount of soluble protein after applying ALS inhibitors has been demonstrated [15]. It has been proposed that this increased free amino acid pool reflects a rise in protein turnover as a result of increased degradation and reduced synthesis rates [16]. Indeed, although protein synthesis occurs following ALS inhibitor treatment, the amino acid components of these proteins are not generated from newly incorporated nitrogen [12] but are instead primarily scavenged from protein degradation. This observation supports the hypothesis that proteases might be involved in protein degradation to release amino acids that cannot be synthesized.

Plants produce hundreds of proteases that are involved in numerous biological processes. The ubiquitin/proteasome system is a major pathway for the degradation and processing of damaged proteins. The 26 S proteasome is a large, multi-subunit protease found in the cytosol and nucleus [17]. In this proteolytic pathway, proteins are first modified through covalent conjugation with ubiquitin, which marks them for rapid hydrolysis by the 26 S proteasome. The 26 S proteasome exhibits caspase-like (peptidylglutamyl peptide hydrolase-like, PGPH), trypsin-like and chymotrypsin-like activities [18]. Other proteases are located in lytic vesicles or the vacuole, such as vacuolar-processing enzymes (VPEs) and papain-like cysteine proteases (PLCPs). VPEs are cysteine proteases that show YVADase activity, cleaving after Asn and Asp [19], [20]. Additionally, serine proteases are involved in immune responses, stomatal density regulation, detoxification and secondary metabolism [21].

Based on the above information, we propose that there is increased protease activity upon herbicide treatment for two reasons: proteases are often induced during stress, and second, because we suspect that proteases are involved in the release of free amino acids upon herbicide treatment.

Here, we characterized the activities of different proteases in pea roots upon treatment with the ALS inhibitor imazamox (Imx) and the EPSPS inhibitor Gly. The activities of major classes of proteases were monitored using fluorescent substrates and through protease activity profiling, showing that the activities of several proteases (e.g., VPEs) were downregulated, whereas the activities of others were upregulated upon treatment with both herbicides.

## Materials and Methods

### Plant Material and Treatment Application

*Pisum sativum* L. cv. Snap Sugar Boys plants were grown in aerated hydroponic culture in a growth chamber as previously described [6]. When the plants were 12 days old, herbicide treatments were applied to the nutrient solution. The ALS-inhibiting herbicide Imx (commercial formula Pulsar®40, BASF Española SA, Barcelona, Spain) was applied at a final concentration of 0.016 mM (5 mg l^−1^). The EPSPS-inhibiting herbicide Gly (commercial formula Roundup®Plus, MONSANTO Agricultura España SL, Madrid, Spain) was applied at a concentration of 0.234 mM (53 mg l^−1^).

For analytical purposes, root samples were collected at different time points after treatment. At harvest, root samples were immediately frozen in liquid nitrogen and stored at −80°C until further use.

### Amino Acid Content Determination

Extraction of amino acids was performed in HCl. Following protein precipitation, the amino acid concentrations in the supernatants were measured using a capillary electrophoresis apparatus equipped with a laser-induced fluorescence detector, as previously described [9].

### Protease Assays and Soluble Protein Determination

Proteins were extracted from 350 mg of ground pea roots in 700 µl of extraction buffer containing 50 mM Tris-HCl (pH 7), 1 mM EDTA, 5 mM DTT, 5 mM β-mercaptoethanol and 1% PVPP following a previously described method [22] with some modifications. The samples were centrifuged for 15 min at 15,000 g at 4°C, and the supernatant was used in protease assays and subjected to protein content determination. The protein concentration was determined as previously described [23].

Protease activities were measured using specific fluorogenic substrate (see below). All fluorogenic substrates were purchased from Sigma-Aldrich (St. Louis, MO, USA), except Val-Arg-Pro-Arg-AMC (VRPR-AMC), which was purchased from Bachem (Bubendorf, Switzerland). All substrates were dissolved in DMSO and diluted in water to their final concentrations. For all examined activities, the assay mixtures contained the specific substrate and the protein extract in a reaction cocktail. Protease activities were measured at 30°C using a Sinergy™ HT microplate reader (BioTek® Instruments Inc., VT, USA) under 380 nm excitation/460 nm emission. The obtained activities were expressed as RFU min^−1^ mg^−1^ protein.

The specific substrates Leu-Leu-Val-Tyr-AMC (LLVY-AMC), Gly-Lys-Arg-AMC-HCl (GKR-AMC-HCl) and Leu-Leu-Glu-AMC (LLE-AMC) were used to evaluate the chymotrypsin- (adapted from [22]), trypsin- (adapted from [24]) and PGPH-like activities (adapted from [25]) of the proteasome, respectively. These three types of activity of the proteasome were determined as the amount of activity inhibited after incubating a crude extract with the proteasome inhibitor MG132 (100 µM final concentration) at RT for 30 min before measuring the enzymatic activities.

YVADase activity was determined using the specific substrate Tyr-Val-Ala-Asp-AMC (YVAD-AMC), which is the most common substrate of caspase 1-like activity, as previously described [26]. YVADase activity as determined in the presence or absence of a VPE inhibitor (I440), which was added at a concentration of 50 µM to the sample, followed by incubation at RT for 30 min prior to the determination of enzymatic activity. For cysteine proteases, the specific substrate Z-Gly-Gly-Arg-AMC (Z-GGR-AMC) was used, as previously described [24]. To detect metacaspase 9-like activity, the specific substrate Val-Arg-Pro-Arg-AMC (VRPR-AMC) was employed, as described elsewhere [27].

### Ubiquitin Western Immunoblotting

Total protein was isolated from the roots as previously described [6]. Western blotting was performed according to standard techniques. A ubiquitin antibody (Ubq11) from Agrisera (Vännäs, Sweden) was used in this assay at a dilution of 1∶5,000. Anti-Rabbit IgG Alkaline Phosphatase (Sigma-Aldrich, St. Louis, MO, USA) was employed as the secondary antibody at a dilution of 1∶20,000, and of the obtained bands were visualized using the Immun-Blot Amplified AP Assay, Bio-Rad 170 6412 (Bio-Rad, Hercules, CA, USA).

### Labeling, Activity-based Protein Profiling (ABPP) and Detection

A 100 mg sample of ground pea roots was labeled. The protein concentration was measured using the RC/DC Protein Assay (Bio-Rad, Hercules, CA, USA) according to the manufacturer’s instructions. For ABPP of the proteasome and PLCPs, MV151 labeling was performed by incubating ∼100 µg of protein in 50 µl of 50 mM PBS buffer (pH 7.5 for the proteasome) or sodium acetate (pH 6.0 for the PLCPs) containing the probe MV151 at 0.4 µM for 1 h, as previously described [28]. For ABPP of VPEs, AMS101 labeling was conducted by incubating ∼100 µg of protein in 50 µl of 400 mM sodium acetate buffer (pH 5) containing 4 mM TCEP and the probe AMS101 at 1 µM for 2 h, as described elsewhere [29]. For ABPP of serine proteases, FPRh labeling was carried out by incubating ∼100 µg of protein in 50 µl of 50 mM PBS buffer (pH 7.5) and the probe FPRh at 2 µM for 1 h, as previously described [21]. Equal volumes of DMSO were added to the no-probe controls. All labeling reactions were conducted in the dark at room temperature.

To achieve protease labeling in competition assays, the control extracts were pre-incubated with the inhibitor for 30 min at room temperature (RT) prior to labeling. Inhibitors and final concentrations used were: E64, cysteine proteases inhibitor 10 µM; MG132, cysteine proteases and proteasome inhibitor 10 µM; epoxomicin, specific proteasome inhibitor 20 µM; I440, VPEs inhibitor 50 µM and Ac-YVAD-CMK, YVADase inhibitor 50 µM. All inhibitors were purchased from Calbiochem (San Diego, CA, USA).

The reactions were terminated following the addition of 20 µl of 4X SDS electrophoresis gel loading buffer (280 mM SDS, 400 mM Tris, 40% glycerol, 1.4 M β-mercaptoethanol and 0.6 mM Bromophenol Blue, pH 6.8). The obtained proteins were separated in 12% SDS polyacrylamide gels. The gels were then washed three times for 15 min with distilled water, and the blots were scanned using a Typhoon 8600 scanner (GE Healthcare, Munich, Germany). The excitation wavelength was set to 532 nm, and emission was measured using a TAMRA filter (580 nm). A GS-800 densitometer (Bio-Rad, Hercules, CA, USA) was used for band quantification.

### Statistical Analysis

The mean values were calculated using the samples obtained from individual plants as replicates. The results were subjected to a separate one-way ANOVA for each day of treatment (SPSS 18.0). The means were separated using the least significant difference method (p<0.05, Fisher protected). Significant differences between each treatment and the control plants (non-treated plants) are highlighted in the figures using a different symbol for each treatment.

## Results and Discussion

### Effects on Growth and the Contents of Free Amino Acids and Soluble Proteins

**Figure 1:**
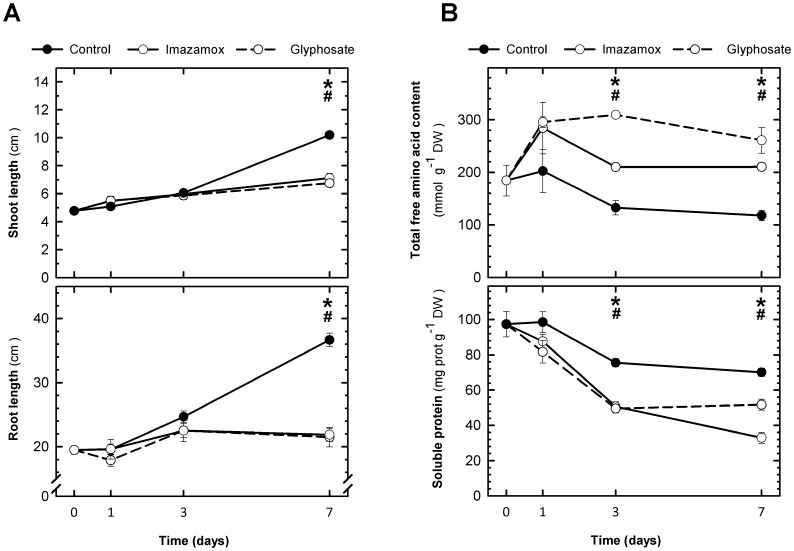
Effect of amino acid biosynthesis inhibitors on growth and amino acid and protein contents. (A) Shoot and root growth of control pea plants and plants treated with imazamox or glyphosate. Means ± SE (n = 8). The symbols indicate significant differences between the control and imazamox (*) or glyphosate (#) treatment on a given day (p<0.05). (B) Total free amino acid and soluble protein contents in the roots of control pea plants and plants treated with imazamox or glyphosate. Means ± SE (n = 4–8). The symbols indicate significant differences between the control and imazamox (*) or glyphosate (#) treatment on a given day (p<0.05).

We previously showed that application of 0.234 mM Gly to pea roots in a hydroponic system results in slow, robust and synchronous death of this crop plant within 20 days [7]. When we tested different Imx concentrations we found that at 0.016 mM Imx have similar effects on the intensity and rate of pea growth retardation as 0.234 mM Gly (Fig. 1A). Both treatments caused arrest of root and shoot elongation, which was significant on the seventh day. In both cases, plant death occurred at 20 days.

We next evaluated the free amino acid and soluble protein contents at 1, 3 and 7 days after treatment (Fig. 1B). While no changes were detected after 24 h, the application of Imx or Gly caused an increase in the total free amino acid content in the roots and a consistent decrease in the soluble protein content from day 3 onward (Fig. 1B). These effects were consistent with the results reported in the literature [7], [9]–[15], suggesting the existence of altered nitrogen metabolism following ALS or EPSPS inhibition. Furthermore, increased free amino acid levels and decreased protein levels suggest that proteolytic activities are increased upon herbicide treatment.

In order to find which proteolytic processes are most significant to the change of free amino acid and amount of soluble protein, it was planned to evaluate free amino acid and soluble protein in herbicide treated plants pre-incubated with proteasome and YVAD-CHO inhibitors as it has been reported before [20], [24], [30]. The objective was to determine if the toxicity of the herbicides was enhanced or alleviated by inhibiting the YVADase activity (with YVAD-CHO) or the proteasome (with MG132). Unfortunately, at day 3 of treatment no differences in the YVADase or the proteasome activities were found between control and pretreated plants and the results were inconclusive.

We next evaluated the activities of the main proteolytic systems found in plants with both herbicides. For these analyses, the time point of 3–4 days following herbicide treatment was selected, representing the shortest time period at which significant differences in both the amino acid and soluble protein contents were observed without any effects on growth (Fig. 1A).

### Effect of Herbicide Treatments on the Proteasome

**Figure 2:**
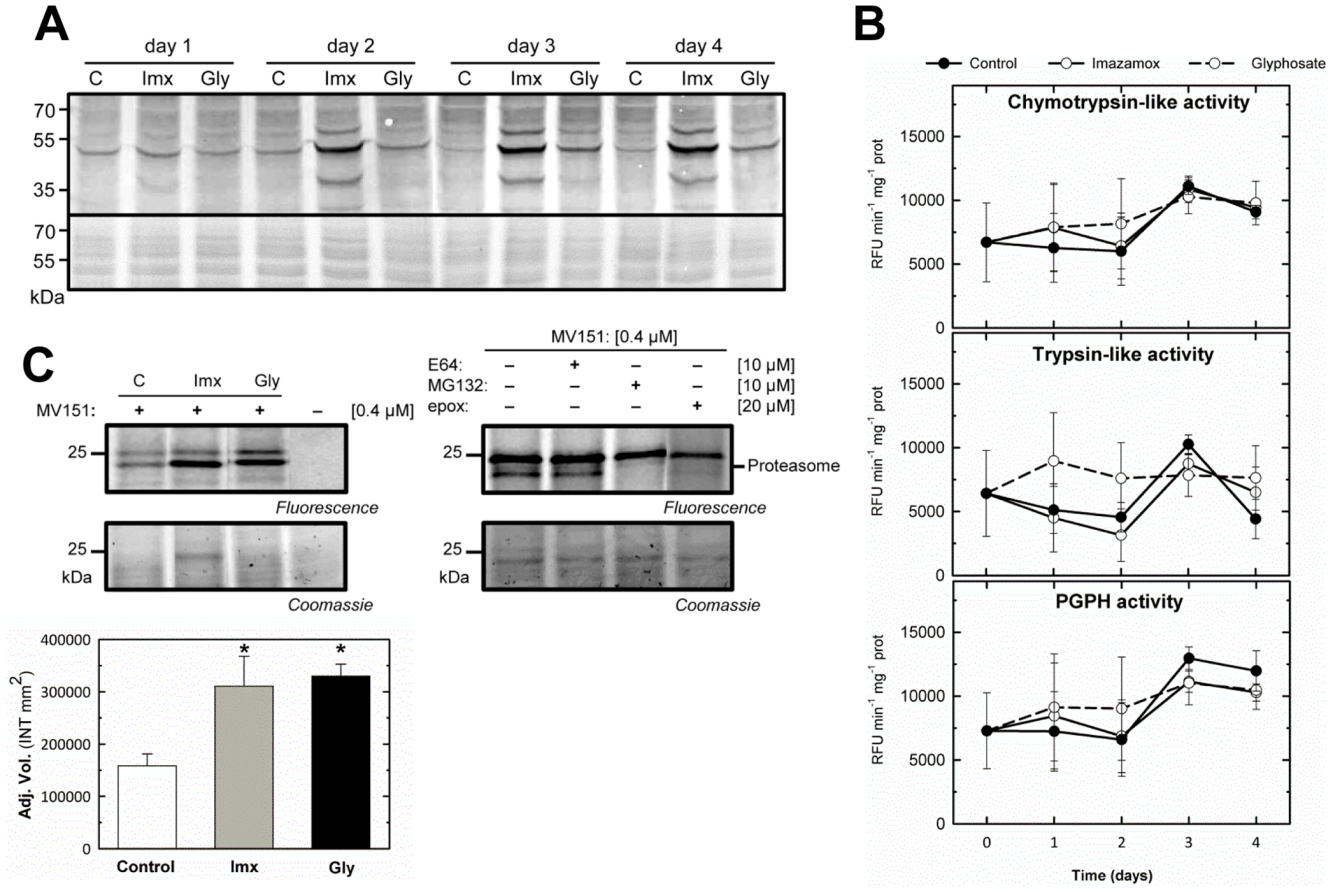
Effect of amino acid biosynthesis inhibitors on the proteasome. (A) Ubiquitin immunoblotting of total protein extracts from control pea plants (C) or plants treated with imazamox (Imx) or glyphosate (Gly) for 1, 2, 3 and 4 days. A total of 30 µg of protein was loaded into each well. The Coomassie-stained protein gel on the bottom shows the total amounts of input proteins. (B) Chymotrypsin-like, trypsin-like and PGPH activities of the proteasome. These activities were measured using the specific substrates LLVY-AMC, GKR-AMC-HCl and LLE-AMC, respectively. The presented data correspond to the activities after subtracting the values obtained following incubation with the proteasome inhibitor MG132 for 30 min from those recorded without MG132. Means ± SE (n = 3). (C) ABPP of the proteasome. Left, a comparison of the labeling profiles observed in pea roots treated with imazamox or glyphosate at day 3 following incubation with 0.4 µM MV151 for 1 h at pH 7.5. Right, a competitive assay in which root extracts were pre-incubated with 10 µM E64, 10 µM MG132 or 20 µM epoxomicin for 30 min before labeling. The results of the competitive assay showed that the 25 kDa signal corresponded to the proteasome. Fluorescently labeled proteins were detected from protein gels via fluorescence scanning. The signals were quantified using a densitometer, and the results are shown in the bar graph. Means ± SE (n = 3). The symbol *indicates significant differences between the control and treatments (p<0.05). The Coomassie-stained protein gel on the bottom shows the total amount of input protein.

The ubiquitin-proteasome proteolytic system plays an important role in eukaryotic cell growth, development and stress responses and the environmental adaptation of plants through the degradation of short-lived and abnormal proteins [31], [32]. The role of the proteasome following herbicide treatment was evaluated through the quantification of polyubiquitinated proteins and proteasome activities (Fig. 2). Ubiquitin immunoblotting of total protein extracts from plant roots was performed 1, 2, 3 and 4 days after herbicide application. Figure 2A shows that there was an increase in the amount of polyubiquitinated proteins upon treatment with both herbicides. This increase was significant at 2 days after Imx and 3 days after Gly treatment, and the signal was stronger following Imx treatment. An increase in the level of ubiquitin-protein conjugates has been reported following other types of abiotic and biotic stresses, such as heat shock [33], darkness, UV radiation, starvation, enhanced ozone levels [34] and cadmium stress [25], [35].

The 26 S proteasome is a large proteolytic complex exhibiting three different types of cleavage activity: chymotrypsin-, trypsin- and caspase-like (PGPH) activities. Fluorogenic substrates were used to determine these activities of the proteasome upon herbicide treatment. Specific fluorogenic substrates are available to monitor chymotrypsin-, trypsin- and caspase-like (PGPH) activities, but these substrates are also subject to proteolytic processes that are not involved in the proteasome. Root extracts were incubated with or without MG132, a widely used proteasome inhibitor that inhibits all three enzymatic activities in proteasome at the concentration used in this experiment [36]. Then, the extracts were subsequently incubated with synthetic peptide substrates conjugated with the fluorescent reporter 7-amino-4-methylcoumarin (AMC). Therefore, the chymotrypsin-, trypsin- and caspase-like (PGPH) activities involved in the proteasome were calculated by subtracting values of activities obtained in the condition with MG132 (activities not involved in the proteasome) from that without MG132 (total activities) [25]. No general effect of Imx or Gly treatment on the chymotrypsin-, trypsin- and caspase-like (PGPH) activities of the proteasome could be identified (Fig. 2B). However, MG132 inhibits not only the proteasome, but also cysteine proteases [37], and thus, the activities estimated following incubation with this inhibitor might not reflect proteasome activity alone.

To overcome this limitation, we used activity based protein profiling (ABPP) to directly detect the active proteasome. ABPP is based on the use of fluorescent small molecule probes that covalently react with active site residues at enzymes resulting in an irreversible labeling that can be detected and quantified form protein gels scanned for fluorescence [38]. We used MV151 [28] to monitor proteasome activity 3 days after herbicide treatment (Fig. 2C). Following labeling with the MV151, two fluorescent signals were detected at 25 kDa. Previous studies involving labeling with the MV151 in *Arabidopsis* leaves revealed three strong fluorescent signals at approximately 25 kDa, representing the three proteasome catalytic subunits [28]. However, we detected only two signals in pea roots (Fig. 2C), similar to the results of proteasome labeling in *Nicotiana benthamiana* [29]. To confirm the identities of these bands, a competitive assay was performed. In this assay, control root extracts were pre-incubated with the inhibitors E64 (which inhibits cysteine proteases, but not the proteasome [39]), MG132 (which inhibits both the proteasome and cysteine proteases) and epoxomicin (which inhibits only the proteasome [18]), followed by labeling with MV151. The observed competition of MV151 labeling with epoxomicin and MG132, but not E64 indicates that the lower signal detected at 24 kDa represents the proteasome (Fig. 2C, right). Importantly, this band showed a 2-fold greater intensity after treatment with Imx or Gly, indicating an increase in proteasome activity following herbicide treatment. Previous studies using the MV151 probe have observed an increase in proteasome activity during defense [28]. At this stage, it is unclear what the upper 25-kDa-band represents, as this band was not competed with E64 or epoxomicin.

The accumulation of ubiquitinated proteins, together with increased putative proteasome activity was observed through ABPP, indicating a role for the proteasome upon herbicide treatment. Accumulation of ubiquitinated proteins has typically been described in association with a concomitant decrease in proteasome activity [25], [31], [35], [36]. Nevertheless, our results demonstrated increases in both proteasome substrate levels and activities. Thus, the herbicide-induced stress on the proteome might result in the accumulation of ubiquitinated proteins, despite the increased proteasome activity, or the increased availability of the substrate might induce proteasome activity. A previous study using phosphinothricin, another type of herbicide that inhibits amino acid biosynthesis, also revealed a role for the proteasome pathway in the response to herbicide treatment [40]. The authors observed that *Arabidopsis* mutant plants with impaired 26 S proteasome function showed increased tolerance to phosphinothricin, suggesting a relationship between the proteasome and changes in the phytotoxic response induced through herbicide treatment.

### Effect of Amino Acid Biosynthesis Inhibitors on Vacuolar Processing Enzymes

**Figure 3:**
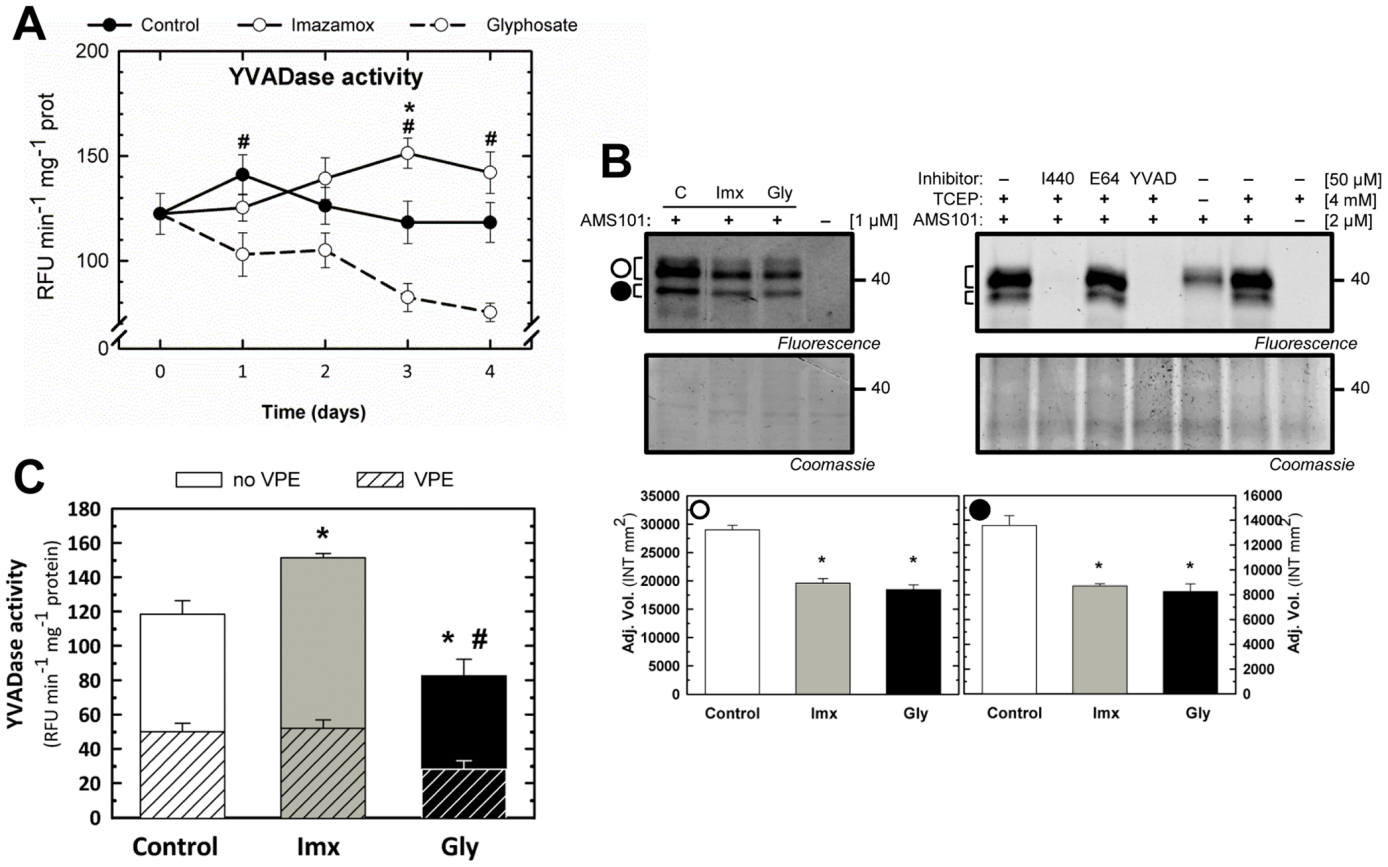
Effect of amino acid biosynthesis inhibitors on the vacuolar-processing enzymes. (A) Total YVADase activity determined using the specific substrate YVAD-AMC. Means ± SE (n = 7). Symbols indicate significant differences between the control and imazamox (*) or glyphosate (#) treatment on a given day (p<0.05). (B) Left, comparison of the VPE labeling profiles in pea roots treated with imazamox or glyphosate at day 3 following incubation 1 µM AMS101 for 2 h. Right, a competitive assay in which root extracts were pre-incubated with the inhibitors E64, Ac-YVAD-CMK (YVAD) and I440 at 10 µM, 50 µM and 50 µM, respectively, for 30 min or extracted without reducing buffer (-TCEP) prior to labeling. Fluorescently labeled proteins were detected in protein gels via fluorescence scanning, and two bands were observed (• and ○). The signals were quantified using a densitometer, and the relative values are shown in the bar graphs. Means ± SE (n = 3). The symbol * indicates significant differences between the control and the treatments (p<0.05). The Coomassie-stained protein gel on the bottom shows the total amounts of input proteins. (C) YVADase activity determined in the presence or absence of the VPE inhibitor I440. YVADase activity is displayed as the total YVADase activity due to VPEs (“VPE”) or without VPEs (“no VPE”). The root extracts were incubated with the inhibitor at a concentration of 50 µM for 30 min at RT prior to adding the specific substrate YVAD-AMC. Means ± SE (n = 3). Symbols indicate significant differences in no-VPE YVADase activity (*) and VPE YVADase activity (#) between the control and treated plants at day 3 (p<0.05).

VPEs, also referred to as legumains, are a family of cysteine proteases that act as asparaginyl endopeptidases [20], [41]. However, Asn is not required at position P1 in the substrate cleavage site, although similar to caspase-like proteases, VPEs cleave after Asp [42], [43]. Both VPE and YVADase activities are involved in programmed cell death (PCD) [20], [41]. VPE activity has also been reported to increase during oomycete infection to mediate protein turnover and nutrient release [29]. The role of VPEs in the mode of action of ALS and EPSPS inhibitors was evaluated through quantification of YVADase activity, and the activity of VPEs was detected through ABPP and assessing the rate of YVADase activity associated with VPEs. A specific fluorogenic substrate was employed to determine YVADase activity upon herbicide treatment. A significant increase in YVADase activity was detected at 3 days after Imx treatment (Fig. 3A). In contrast, a significant decrease in YVADase activity was detected beginning on the first day after Gly treatment (Fig. 3A). ABPP was carried out to directly detect VPE activity, and labeling with the AMS101 probe was performed on day 3 (Fig. 3B). Similar previous labeling studies in *Arabidopsis* leaves have consistently revealed signals at 40 kDa, corresponding to VPEs [29]. Labeling pea root extracts with AMS101 revealed similar signals at 40 kDa (Fig. 3B). To determine whether both detected bands were putative VPEs, a competitive analysis was performed using the following inhibitors: I440 (which contains a Pro-Asp dipeptide and an acyloxymethylketone reactive group and inhibits VPEs), E64 (which inhibits cysteine protease activity, but not caspase-like activity) and YVAD-CMK (which inhibits YVADase activity) (Fig. 3B, right). Importantly, pre-incubation with the VPE inhibitors YVAD-CMK and I440, but not with E64, blocked labeling, suggesting that these signals indeed represented VPEs. In addition, the lack of a reducing agent (no TCEP) also suppressed labeling, consistent with the fact that VPE activity requires reductive conditions [29]. AMS101 labeling of the extracts from herbicide-treated pea roots revealed reduced putative VPE activity upon herbicide treatment (Fig. 3B, left). The quantification of both signals indicated that there was a significant 30% reduction of VPE activity compared to the control (Fig. 3B, down). Notably, these results indicated contradictory patterns between YVADase activity and VPE labeling following herbicide treatment. The observed increase in YVADase activity and decrease in VPE activity suggests that not all YVADase activity is induced through VPEs.

To determine whether the detected YVADase activity was induced through VPEs, a fluorogenic kinetic assay was performed at day 3 with or without the VPE-specific inhibitor (Fig. 3C). Root extracts were pre-incubated with the VPE inhibitor I440 for 30 min at RT, and the YVADase activity in these extracts was subsequently measured and compared with total YVADase activity. Not all YVADase activity was inhibited following pre-incubation with I440 (Fig. 3C), indicating that not all of the observed YVADase activity could be attributed to VPEs. Consistent with the reduction of VPE labeling upon Gly treatment, the YVADase activity induced through VPE was also reduced following Gly treatment, explaining the overall decrease of YVADase activity upon Gly treatment. In the case of Imx treatment, no reduction of YVADase activity from VPEs was observed, in contrast to the decreased VPE labeling. Thus, the increased YVADase activity must reflect the activity of proteases other than VPEs.

Notably, the distinct YVADase activity observed upon herbicide treatment suggests that YVADase activity might play different roles in Imx- and Gly-treated plants. As both an increase and a decrease of YVADase activity were detected following treatment with lethal doses of herbicides, a clear role for YVADase activity in herbicide toxicity cannot be proposed. Although YVADase activity has been implicated in PCD, herbicides inhibiting amino acid biosynthesis have not been reported to induce PCD in treated plants, making it difficult to associate the increase or decrease of YVADase activity with changes in PCD.

### Effect of Amino Acid Biosynthesis Inhibitors on Other Proteases

**Figure 4:**
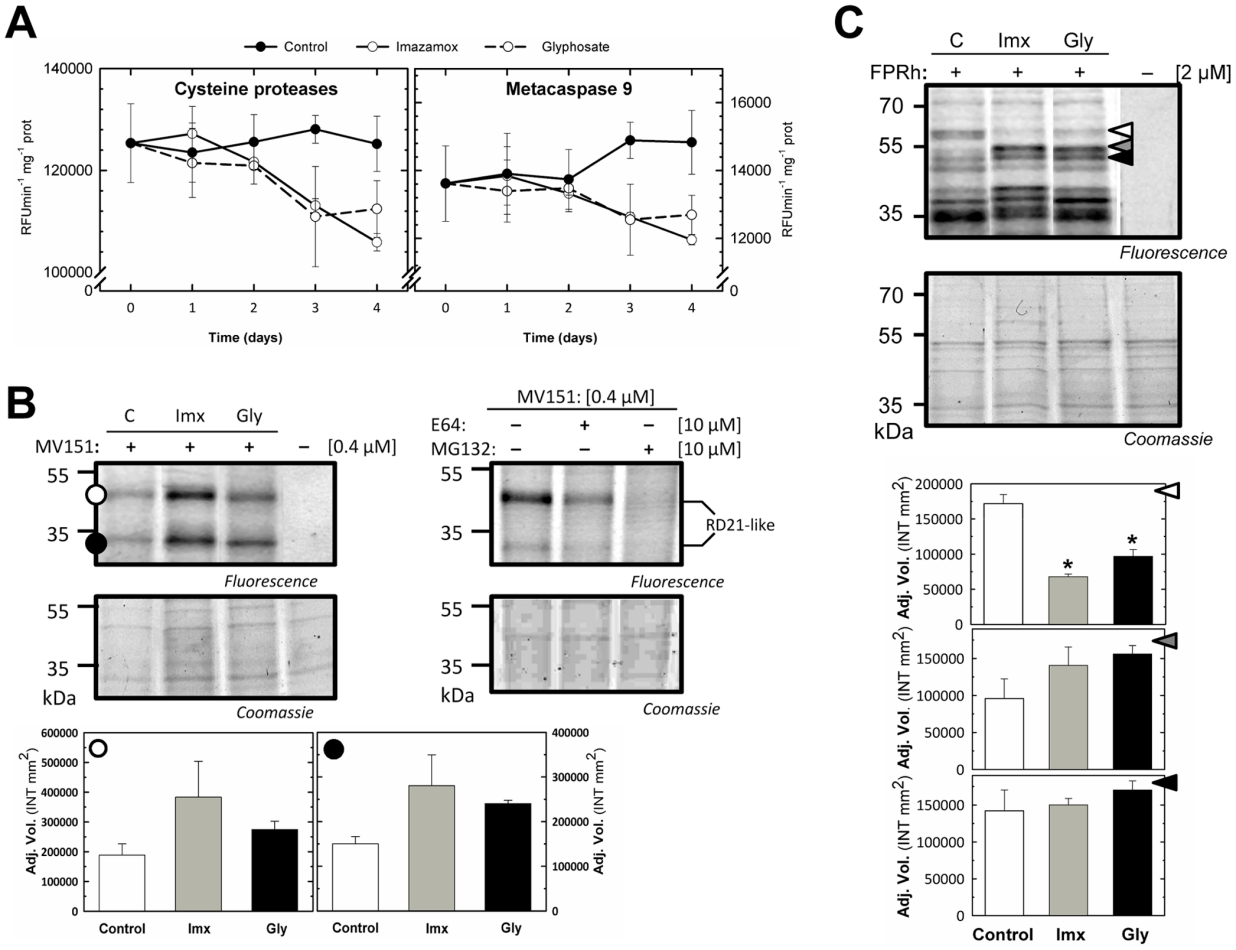
Effect of amino acid biosynthesis inhibitors on other proteases. (A) Cysteine protease and metacaspase 9 activities assayed using the specific substrates GGR-AMC and VRPR-AMC, respectively, in pea roots treated with herbicides that inhibit amino acid biosynthesis. Means ± SE (n = 3). (B) Left, the labeling profile of PLCPs in pea roots treated with imazamox or glyphosate at day 3 following incubation with 0.4 µM MV151 for 1 h at pH 6.0. Right, a competitive assay in which root extracts were pre-incubated with the inhibitor E64 or MG132 at a concentration of 10 µM for 30 min prior to labeling. The 30–40 kDa signals correspond to RD21 PLCPs. Fluorescently labeled proteins were detected in protein gels via fluorescence scanning, and two bands were observed (• and ○). The signals were quantified using a densitometer, and the relative values are shown in the bar graph. Means ± SE (n = 3). The symbol * indicates significant differences between the control and the treatments (p<0.05). The Coomassie-stained protein gel on the bottom shows the total amounts of input proteins. (C) Labeling profile of serine proteases in pea roots treated with imazamox or glyphosate at day 3 following incubation with 2 µM FPRh for 1 h at RT in the dark. Fluorescently labeled proteins were detected in protein gels via fluorescence scanning, and three bands were detected (white, black and grey left-facing triangles). The signals were quantified using a densitometer, and the relative values are shown in the bar graph. Means ± SE (n = 3). The symbol (*) indicates significant differences between the control and the treatments (p<0.05). The Coomassie-stained protein gel on the bottom shows the total amounts of input proteins.

Other proteases (cysteine proteases, metacaspase 9, PLCPs and serine proteases) were evaluated to determine whether their activities were affected by herbicide treatment (Fig. 4).

Cysteine protease and metacaspase 9 activities were monitored using specific fluorogenic substrates in plants treated with Imx or Gly for 0, 1, 2, 3 and 4 days. The activity of both proteases was reduced upon treatment with both herbicides for 3 days (Fig. 4A). Several studies have shown increases in the activities of both of these proteases following stress treatment. Cysteine proteases are induced during oxidative stress and pathogen attack [44]. Metacaspases, such as metacaspase 8, are induced in response to stresses, and *Arabidopsis* metacaspase knockout mutants exhibit increased tolerance to methyl viologen [45]. It has recently been reported that different types of metacaspases act as positive or negative regulators of cell death [46].

ABPP was used to evaluate the activities of PLCPs upon herbicide treatment. Pea root extracts were labeled with the MV151 at pH 6. At this pH, MV151 labels PLCPs at 30 and 40 kDa [28]. After labeling with MV151, two bands were detected, at 30 and 40 kDa in the pea root extracts (Fig. 4B). Labeling is blocked upon pre-incubation with the PLCP inhibitors E64 and MG132, suggesting that the detected bands are PLCPs [28]. Interestingly, these bands showed stronger signals following treatment with Imx or Gly, suggesting that induction of PLCPs is another common effect of herbicide treatment.

Serine proteases are the largest class of plant proteases. These enzymes have been implicated in PCD and seed development and might play a role in the formation of plant secondary metabolites [47]. To determine whether serine protease activities were affected by herbicide treatment, an ABPP experiment using the FPRh probe was performed at day 3. Previous studies conducted using FP-based probes have identified the activities of over 50 *Arabidopsis* serine hydrolases, including subtilases, serine carboxypeptidases (SCPLs) and prolyl oligopeptidases [48]. In the present study, the major differences detected corresponded to three protein bands observed at approximately 55–70 kDa: one was significantly reduced, while the other two were increased following herbicide treatment (Fig. 4C). These different signals most likely represent subtilases (family S8) or SCPLs (family S10), as subtilases are typically 70 kDa, while SCPLs are 50 kDa in size. More experiments are required to confirm the identity of these signals. Nevertheless, while one of the putative serine protease bands was less intense following herbicide treatment, the other two bands were induced in the treated roots. Given that these putative serine proteases exhibit different patterns, it will be interesting to identify these bands through mass spectrometry.

### General Conclusion

An increase in the amino acid content and decrease in the soluble protein content decrease are very well-known effects of herbicides inhibiting amino acid biosynthesis. The increased amino acid pool is thought to be derived from a rise in protein turnover, suggesting that proteases might be involved in protein degradation to provide plants with amino acids that cannot otherwise be synthesized due to herbicide inhibition. This study evaluated whether these increased free amino acid and decreased soluble protein contents were associated with major proteolytic activities. Moreover, we evaluated whether the detected changes were common to the two types of herbicides. This study is the first to evaluate and compare the effects of ALS and EPSPS inhibitors on proteolytic activities.

Theoretically, to explain the increased free amino acid pool, a general increase in proteolytic activity would be expected following herbicide treatment. We detected changes in almost all of the proteolytic systems tested following herbicide treatment. Some of the evaluated proteolytic systems were induced, while others were inhibited after treatment with Imx or Gly. Notably, most of the detected changes were common to both herbicides: proteasome and PLCP activities were induced; VPE activity was decreased; and cysteine protease and metacaspase 9 activities were reduced following both Imx and Gly treatment. Moreover, the pattern of serine proteases displayed several unidentified signals that similarly increased or decreased following treatment with both herbicides. The only observed difference between the effects of the two herbicides was in terms of YVADase activity, where an increase under Imx but a decrease under Gly treatment was observed.

New pesticides are currently being developed to replace compounds that no longer meet environmental or toxicological safety requirements. However, the battle against the development of weed resistance will require additional compounds that mimic herbicides but inhibit different biochemical targets to alleviate selection pressure [49], [50]. In this context, plant proteases might represent potential new targets for herbicide development, and understanding their roles in the mode of action of herbicides could facilitate the development of new compounds with herbicidal effects [47].

The similar profiles of the proteolytic activities detected following ALS and EPSPS inhibition indicate the important role of proteolysis. Nevertheless, as some of the evaluated proteolytic systems were induced while others were reduced following the application of lethal concentrations of herbicides, the specific role of proteolytic activities in the toxicity provoked by these herbicides cannot be defined. Further pharmacological and genetic experiments are therefore required to elucidate the physiological implications of the changes in proteolytic systems observed in the presence of herbicides inhibiting amino acid biosynthesis.
